# Two Demosponges as Promising Bioremediators of a Potential Pathogenic *Vibrio*

**DOI:** 10.3390/biology14020140

**Published:** 2025-01-29

**Authors:** Joseba Aguilo-Arce, Maria Scrascia, Roberta Trani, Carlo Pazzani, Pere Ferriol, Caterina Longo

**Affiliations:** 1 Department of Bioscience, Biotechnology and Environment, University of Bari Aldo Moro, Via Orabona 4, 70125 Bari, Italy; joseba.aguiloarce@uniba.it (J.A.-A.); maria.scrascia@uniba.it (M.S.); carlo.pazzani@uniba.it (C.P.); caterina.longo@uniba.it (C.L.); 2 Interdisciplinary Ecology Group, Department of Biology, University of the Balearic Islands, Carretera de Valldemossa km 7.5, 07122 Palma de Mallorca, Spain; pere.ferriol@uib.cat

**Keywords:** porifera, bioremediation, clearance rate, retention efficiency, ampicillin-resistance, antimicrobial resistance, *Vibrio parahaemolyticus*

## Abstract

Marine sponges play a fundamental role in the proper functioning of the ecosystem by filtering organic matter and contributing to nutrient fluxes. These animals have been proposed as efficient bioremediators of microbiological contamination in various environmental conditions subjected by anthropogenic pressure. In the present study, the bioremediation potential of the demosponges *Aplysina aerophoba* and *Geodia cydonium* was analyzed ex situ. For this purpose, the viable count of an antibiotic-resistant bacterial strain belonging to the species *Vibrio parahaemolyticus* was assessed in presence of the selected sponge species. Although some sponge individuals showed closed oscula during the first hours of the experiment, *A. aerophoba* and *G. cydonium* reduced the bacterial load in the seawater up to five orders of magnitude in 72 h. In addition, they had high clearance rates and retention efficiencies, with almost complete removal of the tested bacteria. Low *Vibrio* concentrations were observed in all tanks after six days, suggesting no excretion of viable *Vibrio* from sponges. These results corroborate the usefulness of *A. aerophoba* and *G. cydonium* as bioremediators of bacteria and therefore appear to be ideal candidates for bioremediation purposes in anthropogenic environments, such as aquaculture facilities, where multidrug-resistant bacteria may play a role in the spread of antimicrobial resistance.

## Simple Summary

Marine sponges, widespread across the world’s oceans, are highly efficient filter feeders capable of retaining various organic particles, including bacteria. In this study, we evaluated the capacity of two common Mediterranean sponge species to filter and retain a multidrug-resistant strain of *V. parahaemolyticus*. Both sponges demonstrated high clearance and retention efficiencies, with no bacterial excretion observed after six days. The sponges remained healthy throughout the experimental period, showing no signs of infection. These findings highlight the sponges’ resilience and effective filtering capabilities, emphasizing their potential role in impacted environments such as aquaculture systems by mitigating the spread of antimicrobial resistance—a growing global challenge.

## 1. Introduction

With more than 9600 valid species worldwide [1], sponges are one of the most abundant benthic invertebrates on the planet, colonizing different types of habitats at a wide range of depths [2]. They are capable of filtering and retaining a broad spectrum of organic particles, including viruses, bacterioplankton, picoflagellates, and microphytoplankton (0.1–50 µm) [3,4,5]. Moreover, in recent years, it has become increasingly evident that many sponges utilize dissolved organic matter (DOM) as their main food source [6]. Their metabolism encompasses the excretion of inorganic nutrients [7], supporting their role as key players in maintaining ecosystem balance and functionality. Recent studies highlight the interaction between sponges and cycles of dissolved organic and inorganic nutrients, leading to substantial net fluxes. For instance, their unique metabolism and symbiotic relationships are responsible for the conversion of nitrogen and phosphorus compounds, which, in certain environments, strongly support phytoplankton and bacterioplankton communities [8]. This recirculation of nutrients through marine sponges is called the “sponge loop” [9,10]. Due to their continuous interaction with the water column, sponges serve as effective bioindicators of marine pollution [11,12,13,14,15].

Sponges capture particles at three functional sites based on size: large particles (>50 µm) are taken up by epithelial pinacocytes at the surface, smaller particles (<50 µm) are captured by pinacocytes in the canal walls, and the smallest particles (<5 µm) are trapped in choanocyte chambers [16]. Captured particles are transferred to mesohyl cells via transcytosis and phagocytosed by archaeocytes. Research indicates that sponges are highly efficient but largely non-selective in particle uptake, with particle size being a key factor [5,16,17].

Among microparticles consumed by sponges, bacteria constitute a fundamental energy source [17,18], making these animals important candidates for the bioremediation of microbial pollution associated with wastewater. Near mariculture facilities, for example, eutrophication resulting from the high amount of organic matter creates optimal conditions for the proliferation of bacteria such as Vibrionaceae, which can be detrimental to farmed fish, thus generating significant losses for the sector [19,20].

Among potentially pathogenic bacteria, *Vibrio parahaemolyticus* is a natural bacterium in marine and estuarine environments and is often found in seafood, particularly shellfish [21,22]. Given its common presence, it is important to understand how it interacts in aquaculture systems, where conditions can promote its proliferation and its potential spread to human consumers. This bacterium is one of the leading causes of seafood-related gastroenteritis in humans. The pathogenic strains of *V. parahaemolyticus* can cause serious health issues, making it critical to monitor how aquaculture practices may influence its prevalence, pathogenicity, and resistance patterns. The antimicrobial resistant (AMR) variant of *V. parahaemolyticus* is of particular concern [23]. Aquaculture often involves the use of antibiotics to control infections, which can promote the development of AMR bacteria [24]. Studying AMR variants helps assess the risks of antibiotic use in aquaculture and evaluate how such practices might contribute to the spread of resistant strains [25]. The presence of AMR *V. parahaemolyticus* in aquaculture systems poses risks not only to the aquatic environment but also to public health [26]. Indeed, resistant strains can transfer their resistance genes to other bacteria, potentially creating harder-to-treat infections in both animals and humans [27]. By focusing on AMR variants, researchers can better understand the pathways of resistance spread and develop strategies to mitigate its impact.

In this scenario, some studies have focused on understanding and quantifying the filtration capacity of sponges towards potential pathogenic bacteria such as *Vibrio* in both ex situ [28,29,30,31,32,33,34,35,36,37] or in aquaculture facilities [38,39,40,41], and have proposed them as useful bioremediators of these microorganisms. However, the list of organisms used for these trials comprises few species, with *Hymeniacidon perlevis* (Montagu, 1814) being by far the most studied sponge.

The demosponges *Aplysina aerophoba* (Nardo, 1833) and *Geodia cydonium* (Linnaeus, 1767) have been scarcely tested with regard to *Vibrio* filtration. The former is a more studied species, with research focusing on its pumping, filtering and nutrient excretion capacities, e.g., [31,42,43,44,45], while for *G. cydonium* there are no trials that have studied such capabilities. Therefore, due to the lack of information on the filtration capacity of these sponges and the increasing concern about acquired antibiotic resistance of some microorganisms, this study aims to evaluate the ex situ bioremediation potential of *A. aerophoba* and *G. cydonium* against a multidrug-resistant halophilic bacterial strain belonging to the species *Vibrio parahaemolyticus* (family Vibrionaceae), discussing their suitability as biofilters in anthropogenic environments such as open-sea aquaculture facilities.

## 2. Materials and Methods

### 2.1. Specimen Collection

Both *A. aerophoba* and *G. cydonium* individuals came from the REMEDIA Life Integrated Multi-Trophic Aquaculture (IMTA) system [46,47] located in the Maricoltura Mar Grande fish farm (Mar Grande of Taranto, northern Ionian Sea), where sponges were reared around fish cages in rope systems with net bags. Three similar individuals of each species were randomly selected and transported in refrigerated tanks. Once in the laboratory, the individuals were rinsed, cleaned of possible fouling organisms and left to acclimate in two 50 L tanks of constantly aerated artificial seawater for three days. A total of 24 h before the experiment, the water was changed to Artificial Filtered Seawater (AFSW) prepared with commercial aquarium salt (Red Sea Salt, Aquariomania) filtered through 0.22 µm pore size filters (Merck Millipore) to avoid contamination of microorganisms and starve the individuals.

### 2.2. Bacterial Load Preparation

The multidrug-resistant *Vibrio parahaemolyticus* strain CIRPS 4253 from the laboratory collection was used for bioremediation experiment. This strain has demonstrated resistances to several antibiotics (ampicillin, trimethoprim, colistin, carbenicillin, penicillin, lincomycin, bacitracin, cephalexin), among which ampicillin (AMP) was selected to follow and detect the bacterium throughout the experiment. In order to prepare *inoculum* at the final concentration of 10^7^ CFU mL^−1^ in each tank, a viable count assay of the strain CIRPS 4253 was performed by plating serial 10-fold dilutions of an overnight (O/N) culture. The liquid-rich medium Nutrient Broth (OXOID, Milan, Italy) and the differential/selective solid medium Thiosulfate–Citrate–Bile salts–Sucrose agar (TCBS, OXOID, Milan, Italy) both added with NaCl (C_f_ 2%), and AMP (C_f_ 100 µg mL^−1^) were used. Incubation was always performed at 37 °C. The bacterial concentration was calculated in Colony Forming Units (CFU) per mL (CFU mL^−1^) with respect to the volume plated and the selected dilution. *V. parahaemolyticus* colonies were recovered from the selective TCBS medium, and species was confirmed by the API20E system [48].

### 2.3. Experimental Design

Nine experimental tanks (3 L of AFSW each) were set up: three containing *A. aerophoba* explants, three with *G. cydonium* explants, and three control tanks without sponges. Tanks were maintained with continuous aeration, natural light cycles, and at room temperature (22 ± 1 °C). All tanks were inoculated with the bacteria strain CIRPS 4253 at a final concentration of 10^7^ CFU mL^−1^. Water samples (10 mL) were collected from each tank at 0, 1, 3, 8, 24, 48, 72 and 144 h after the inoculation to assess the viable count of CIRPS 4253.

### 2.4. Filtering Activity Estimation

Filtering activity was inferred by bacterial viable count estimated (for each water sample) over time. Tenfold dilutions of the samples (reaching 10^−7^) were plated in TCBS added with NaCl and AMP, as described above. The data were reported as the mean CFU mL^−1^ value ± the standard error (SE) of each experimental tank set. Sponge surface, color and osculum opening served to monitor the well-being of sponge specimens throughout the experiment. After six days (144 h) from inoculation, for *A. aerophoba* and *G. cydonium* explants the volume (V, 47.5 mL ± 1.44 SE and 42.5 mL ± 10.1 SE, respectively), wet weight (WW, 40.84 g ± 2.71 SE and 42.44 g ± 7.57 SE, respectively) and dry weight (DW, 5.44 g ± 0.31 SE and 9.63 g ± 1.68 SE, respectively) were measured using a graduated beaker and a precision scale before and after 24 h at 100 °C, respectively.

At each sampling time, the formula described by Coughlan [49] for clearance experiments was used to estimate the clearance rate (CR) of the sponges, which measures the bacterial removal from the seawater as a function of time (T), volume (V) of water used in the filtering experiment and sponge biomass (dry weight, DW):

(1) $$CR=\frac{\ln(C_{t0}/C_{tx})\times V}{T\times DW}$$

Retention efficiency (R) was calculated as a percentage for the difference in bacterial concentrations with the following equation:

(2) $$R=100\times \frac{C_{t0}-C_{tx}}{C_{t0}}$$

where C_t0_ is the initial bacterial concentration and C_tx_ is the bacterial concentration at each sampling time [50].

### 2.5. Vibrio Parahaemolyticus Detection in Sponge Tissue

At the end of the experimental trial (144 h after the inoculation of *V. parahaemolyticus* CIRPS 4253) and before the biomass measuring, halves of individuals of each sponge species were cut to detect the presence of the bacterial strain inside sponges. After rinsing with AFSW, an extract was obtained by homogenizing the biomass with a mortar and filtering it through a sterile gauze. The crude extract and tenfold dilutions were plated on TCBS (2% NaCl and C_f_ 100 µg mL^−1^ AMP added), and plates were incubated O/N at 37 °C.

Moreover, each crude extract and its O/N culture (100 µL in Tryptic Soy Broth + 2% NaCl) were further subjected to antibiogram assay with effective antibiotics against CIRSP 4253 (chloramphenicol 30 µg, kanamycin 30 µg, nalidixic acid 30 µg, streptomycin 10 µg, tetracycline 30 µg and trimethoprim/sulfamethoxazole 25 µg) and AMP (10 µg), both extracts on Nutrient Agar (OXOID, Milan, Italy) and TCBS added with NaCl (C_f_ 2%). Plates were incubated O/N at 37 °C.

### 2.6. Statistical Analysis

To analyze the variation in bacterial concentration, a linear mixed model was used to assess statistical differences applying a repeated measures analysis of variance (ANOVA), considering treatment (i.e., sponge species or control without sponges) and time factors as fixed effects while each tank was included as random effect (useful to avoid underestimation of variability and provide more accurate estimates of fixed effects). Temporal trends were analyzed by means of the Mann–Kendall trend test [51]. All the statistical analysis was performed using R 4.3.0 (R Foundation, Vienna, Austria).

## 3. Results

### 3.1. Bacterial Load Bioremediation

**Figure 1:**
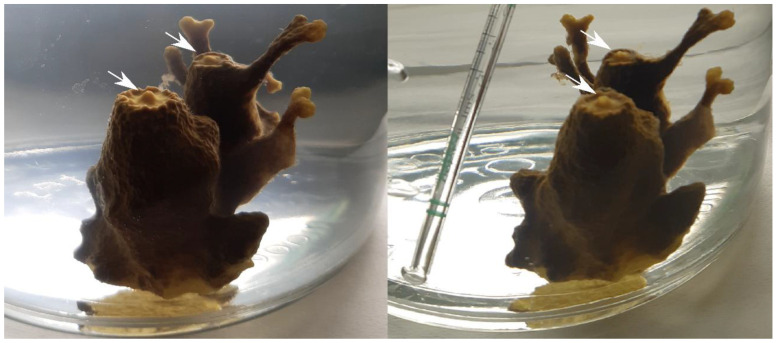
Oscula dynamics of *A. aerophoba* during the experiment. White arrows indicate closed oscula at the beginning of the trial (**left**) and their opening 24 h after the start (**right**).

**Figure 2:**
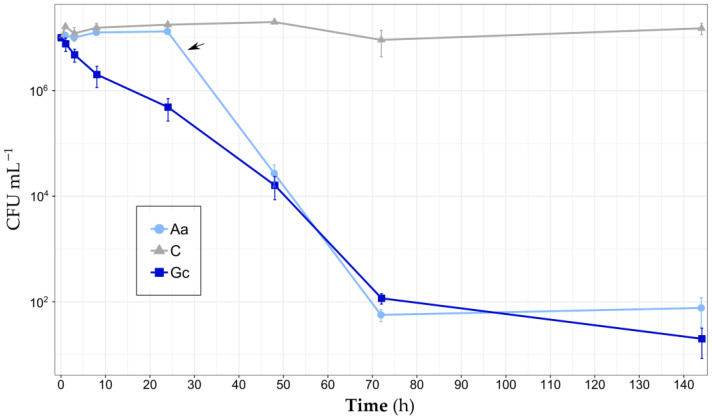
*Vibrio parahaemolyticus* CIRPS 4253 viable concentrations (mean value ± SE) in the seawater throughout the experiment in control (C), *A. aerophoba* (Aa) and *G. cydonium* (Gc) tanks. Arrow indicates the oscula opening in *A. aerophoba* individuals.

Each sponge species showed different responses to acclimatation and starvation. During the clearance trial, *A. aerophoba* kept its oscula closed for 24–48 h (Figure 1), while the other specimens exhibited no visible signs of stress. The concentration of the bacteria strain *V. parahaemolyticus* CIRPS 4253 in *G. cydonium* tanks decreased by 50% within 3 h and remained significantly lower than the control concentrations from onwards (*p* < 0.001). During the first experimental day, bacterial load in *A. aerophoba* and control tanks was statistically similar until 48 h, when a significant decrease was observed (*p* < 0.001), filtering more than 99.97% of the tested strain in less than 24 h (Figure 2). Overall, a significant decreasing trend in bacterial concentration was detected in *G. cydonium* tanks (p < 0.01), unlike in *A. aerophoba* tanks (*p* = 0.57) due to the initially high bacterial load while the oscula were closed. Still, the lowest mean concentrations were recorded 72 h and 144 h after the experiment start in *A. aerophoba* and *G. cydonium* tanks, with values six orders of magnitude smaller (CFU mL^−1^ ± SE: 56.67 ± 14.53 and 20 ± 11.55, respectively). During the last three days of the experiment, low bacterial concentrations remained constant (*p* = 1.00). In the control tanks without sponges, the concentration of *V. parahaemolyticus* CIRPS 4253 increased until 48 h (reaching 1.98·10^7^ CFU mL^−1^ ± 0.1 SE), and from there maintained a mean value of 1.44·10^7^ CFU mL^−1^ (±0.12 SE) without a significant temporal trend (*p* = 0.49).

**Figure 3:**
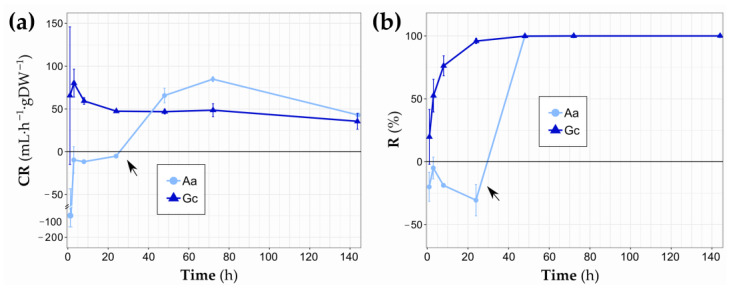
Clearance rate (CR) (mean ± SE) (**a**) and retention efficiency (R) (mean ± SE) (**b**) of *A. aerophoba* (Aa) and *G. cydonium* (Gc) at each sampled time. Arrows indicate the oscula opening in *A. aerophoba* individuals.

Both sponge species effectively filtered the bacterial load in the tanks, albeit with different temporal trends (Figure 3). Due to the closed oscula, *A. aerophoba* showed negative mean clearance rate and retention efficiency for the first 24 h, obtaining both maximum values at 72 h (84.84 mL h^−1^ gDW^−1^ ± 2.88 SE and 99.99%, respectively) and showing significant increasing trends (*p* < 0.001 and *p* = 0.001, respectively). In contrast, the *G. cydonium* tanks displayed positive mean clearance and retention efficiencies after just 1 h (65.52 mL h^−1^ gDW^−1^ ± 80.47 SE and 19.75% ± 21.7 SE), reaching maximum clearance rates at 3 h (80.22 mL h^−1^ gDW^−1^ ± 16.3 SE) and retention efficiency at 72 h (99.99%) from the beginning. The first sampling time showed a very high standard error due to the variability between replicates, but afterwards, clearance rates stabilized at around 50 mL h^−1^ gDW^−1^, showing a decreasing trend from the beginning (*p* < 0.01). Retention exceeded 95% after 24 h, reaching almost 100% at the end of the experiment (increasing trend, *p* < 0.001).

### 3.2. Presence of Vibrio in Sponge Tissues

The presence of viable *Vibrio* in the sponge tissue was evaluated on crude extract of both tested sponges after 144 h from the inoculation of *V. parahaemolyticus* CIRPS 4253. The crude extracts of *G. cydonium* contained 17 CFU gDW^−1^ of AMPR bacteria, while no colonies were observed in *A. aerophoba* extracts. The low abundance of cultivable microbes in the extracts at that time did not allow for the performance of the antibiogram assay. Therefore, overnight incubation of extracts of both species was necessary to allow bacteria to replicate. Still, no visible growth was detected for *A. aerophoba*, but, for *G. cydonium*, the same resistance pattern was observed on both NA and TCBS media, showing sensitivity to all tested antibiotics (see Section 2.5) except ampicillin. This resistance pattern further suggested that the main cultivable microorganism was *V. parahaemolyticus* CIRPS 4253.

## 4. Discussion

In the present study, we demonstrated that the demosponges *Aplysina aerophoba* and *Geodia cydonium* effectively filter and retain a high bacterial load of multidrug-resistant *Vibrio parahaemolyticus* under ex situ conditions. This highlights their potential use as bioremediators in IMTA systems, where they have already been successfully reared [47,52,53,54,55].

In the first 24 h of the experiment, the filtering performance of *A. aerophoba* were influenced by closed oscula. Many sponge species experience intermittent pumping inactivity in response to environmental stressors [56,57,58], which can affect filtration and respiration processes [59,60]. In the case of *A. aerophoba*, it has been shown that environmental factors such as exposure to air, temperature and salinity can negatively affect its pumping capacity, inducing oscula closure that can last for several hours or even days [45,61]. In specimens with a greater number of oscula, the intermittent closure of some of them does not imply a cessation of the individual’s pumping [45]. However, in this study, individuals with two or three osculawere led to a total interruption of pumping. This physiological response may have been influenced by their origin in an anthropogenic environment, such as mariculture, and their acclimation to artificial sea water, the conditions of their starvation period, and its duration. This could also account for the variability in the results observed with *G. cydonium* during the first hour, where one specimen exhibited no signs of filtration (negative values of CR and R in Figure 3). This delay of the filtering activity in ex situ experiments, although shorter and not attributable to closed oscula, has been previously noticed [37].

Nevertheless, both *A. aerophoba* and *G. cydonium* removed *V. parahaemolyticus* strain CIRPS 4253 from the experimental tanks. Retention efficiency values of 99.99% were recorded for both species at the end of the experiment, being similar or higher at different sampling times to values published in time-controlled experiments for other sponge species [35,50,62,63]. Clearance rate values ranged between 47.5 and 80.49 mL h^−1^ gDW^−1^ for *G. cydonium* and −103 and 84.84 mL h^−1^ gDW^−1^ for *A. aerophoba*, with the highest values at 3 h and 72 h, respectively.

When compared to published data (Table 1), the CR observed in this study appear lower than those of other sponge species, probably due to the size of the sponge explants used in the present study and the different microbiological abundance. Both studied species are classified as High Microbiological Abundance (HMA) sponges, which are known to have lower pumping rates than Low Microbiological Abundance (LMA) sponges [64]. Interestingly, for *G. cydonium,* the CR stabilized after 24 h, even when bacterial concentrations were low at the end of the experiment. When compared with other HMA sponge species, *A. aerophoba* showed a higher clearance rate (when oscula opened) than *Chondrilla nucula* Schmidt, 1862, and *Chondrosia reniformis* Nardo, 1847 (between 3 and 12 mL h^−1^ gDW^−1^) [63,65]; in terms of retention rate, the values from this study align with those published previously [31], albeit being two orders of magnitude higher due to the higher initial bacterial concentration used in the present experiment. For *G. cydonium*, the CR was higher than those of *Agelas oroides* (Schmidt, 1864) (between 8 and 32 mL h^−1^ gDW^−1^) [63] but lower than its congener *G. barreti* (96 mL h^−1^ gDW^−1^) [66]. When considering laboratory trials with *Vibrio* spp., both studied species showed similar or slightly higher CRs than *Tethya meloni* Corriero, Gadaleta and Bavestrello, 2015 and *Sarcotragus spinosulus* Schmidt, 1862 (66 and 45 mL h^−1^ gDW^−1^ respectively) [35,36] but lower than the LMA sponge *Halichondria (Halichondria) melanadocia* de Laubenfels, 1936 (~367 mL h^−1^ gDW^−1^) [28]. Conversely, the Heteroscleromorpha *H. perlevis* and the Keratosa *Aplysilla rosea* (Barrois, 1876) demonstrated markedly higher CRs (232 and 537 mL h^−1^ gDW^−1^ respectively) [29,37]. Overall, the CRs found in this study agree with previously reported data and confirm the usefulness of these species as microbial bioremediators.

Sponges are highly efficient, yet largely non-selective filter feeders [5,16,17]. Their ability to filter a wide range of particles, including potentially harmful microorganisms such as *Vibrio* species, makes them particularly relevant for aquaculture systems. These microorganisms, including *V. parahaemolyticus*, pose significant concern in aquaculture due to their role in diseases like vibriosis, which affect both marine organisms and, potentially, humans [22,67]. Although sponges generally do not selectively target specific bacterial taxa, their filtration efficiency is influenced by physiological capacity and particle size [16,68]. The filtration process in sponges is driven by a complex system that involves the structure of the aquiferous system, specialized cells, and the ability to adjust pumping rates based on environmental conditions [4,56,57,64,69,70,71]. This dynamic filtration mechanism enables sponges to continuously process large volumes of water, which is especially important in aquaculture settings, where maintaining water quality and minimizing bacterial load is crucial. The integration of natural biofilters, such as marine sponges, into aquaculture systems, particularly in IMTA systems, offers a promising solution to reduce bacterial loads, decreasing the need for antibiotics and helping to mitigate the spread of AMR bacteria. This approach aligns with the One Health framework, which emphasizes the interconnectedness of human, animal, and environmental health [27].

Beyond bioremediation, sponges provide further advantages in aquaculture systems, both open-water and land-based systems [40,41,72,73], such as contribution to nutrient cycling and sustainable biomass production for compound recovery [38,74,75].

IMTA co-cultivate fed species, such as fish or crustaceans, with extractive organisms like sponges, mussels, polychaetas and seaweeds, which remove organic and inorganic substances, enhancing environmental sustainability [53]. Recent research at the Mediterranean REMEDIA Life IMTA plant has demonstrated the effectiveness of this approach [72]. In such facilities, the potential of extractive species is significant, while the resulting biomass can offer additional economic benefits [53,76,77,78]. In these facilities sponges mitigate dissolved organic matter (DOM), with particulate organic matter (POM) constituting only a minor portion of their intake [6]. As DOM is not a bioavailable food source for most heterotrophic organisms, sponges play a unique and valuable role in IMTA systems. In fed aquaculture, DOM production is inherent and increases significantly with the inclusion of seaweeds as IMTA components [79,80]. This excess DOM can stimulate bacterial growth, including pathogenic strains. Sponges mitigate this microbial load by consuming DOM [6] and removing harmful bacteria [39]. Beyond removing harmful particles like bacteria, viruses, and fecal pellets, sponges enhance productivity by converting DOM into POM (detritus) through the “sponge loop” [9]. This detritus supports benthic food chains and serves as a valuable resource for detritivores, including commercially valuable species like sea cucumbers [81]. Sponges have shown success in both open-water and land-based systems [40,46,62,74]. For instance, in the REMEDIA Life IMTA system, the concentration of *Vibrio* before and after placement of the bioremediators has already been evaluated, with positive effects due to these organisms [72]. The initial values in this environment (~200 CFU/mL) were five orders lower than the initial concentration used in the present work, suggesting that if in this experiment sponges were able to filter out almost the entire bacterial load, the maximum concentration found in a mariculture system should not affect their capabilities. The mechanisms by which sponges process vast amounts of microorganisms while maintaining stable symbiotic communities remain an area for future research. Experimental studies consistently demonstrate reductions in microorganism concentrations across a range of initial levels, supporting the robustness of sponge filtration under varying conditions [35,63].

The long-term incubation of the present work has made it possible to evaluate the filtration and retention capacity of *A. aerophoba* and *G. cydonium* for six days. Already, at 72 h, remarkably low bacterial concentrations were measured, and the water samples collected at the end of the experiment (144 h from the start) from both *A. aerophoba* and *G. cydonium* tanks showed similar values, suggesting that the tested sponges do not expel the filtered *Vibrio*. It has been seen that in filtration experiments on successive days, depending on the species, the filtration capacity [63] is maintained, which could indicate that *A. aerophoba* and *G. cydonium* are capable of filtering even greater amounts of bacteria if they were supplied at three-day intervals (the water column renewal time measured in this experiment). Additionally, our test showed viable AMP^R^ colonies only in the *G. cydonium* crude extract six days after inoculation, at low concentrations (<100 CFU gDW^−1^). During other incubation experiments, the sponge *H. perlevis* can accumulate culturable bacteria at concentrations up to almost four orders of magnitude higher than the initial in 24 h (reaching almost 10^10^ CFU gWW^−1^) [40]. Therefore, low concentrations at the end of the present trial, such as those detected both in the sponge and surrounding water, would suggest digestion of the antibiotic-resistant bacteria [32,33].

It should be noted that symbiotic bacteria of the genus *Vibrio* have previously been identified in both tested sponge species [82,83], specifically one isolate of *G. cydonium* showing high similarity with a strain known to be ampicillin-resistant collected from mariculture environments [84]. However, the sponge microbiome has been found to be stable under mariculture trials of these animals [85,86]. Thus, they are not likely to expel any potentially harmful microorganisms, yet, even so, their contribution to the bacterial load would probably be negligible. In addition, the filtration activity of other bioremediating fouling organisms in IMTA systems, such as polychaetes and ascidians, could also contribute to its mitigation [87,88]. Although sponges are considered largely unselective filter feeders [5,16,17,63], under ex situ conditions *H. perlevis* has shown a selective feeding on pathogenic *Vibrio*, rejecting its filtration in presence of other bacteria [30,32]; moreover, an affected genetic expression was also quantified when sponges were infected by these microorganisms [32]. In the present study, however, explants of both species remained in good condition after six days of exposure to a high bacterial concentration, retaining nearly the entire filtered microbial load without showing any visible damage. This is noteworthy given that mass mortality events in sponges, linked to the proliferation of *Vibrio* species, have been reported in the Mediterranean Sea [89,90]. In this sense, it could be thought that *V. parahaemolyticus*, although pathogenic for other cultured species, e.g., [91,92], is not infectious for the sponges used, making them valuable as bioremediator organisms of these pathogens. In fact, *A. aerophoba* has been found to similarly filter *Vibrio* and other bacteria that might be present in fish farms [31], supporting the latter idea and without restricting the target species of its filtration in aquaculture environments. Nevertheless, further experiments with other bacterial species together with *Vibrio* to test this hypothesis and more detailed analyses (such as histology, electron microscopy or biochemical and genetic tests) would help to better understand the processes of filtration, digestion and the effects that this strain may cause on the tested sponges.

**Table 1:** Ex situ demosponges’ mean clearance rates (CR) according to the species subclass, microbiological abundance (High Microbial abundance, HMA; Low Microbial Abundance, LMA) and explant biomass. CR values for the *A. aerophoba* of the present paper refer to those measured when oscula opened. Numbers (n) next to the names of sponge species indicate the quantity of published CR values for each species. Names in bold relate to experiments with *Vibrio*.

Subclass	MA	Species (n)	Mean CR mL·h^−1^·gDW^−1^ (SE) [min–max]	Mean Biomass gDW (SE) [min–max]	References
Heteroscleromorpha	LMA	*Aaptos spp. (1)*	1872.53 (673.31) [3.57–12,857]	8.19 (2.63) [0.001–34.59]	[93]
		*Axinella cannabina (3)*			[63]
		*Crambe crambe (1)*			[94]
		***Halichondria melanadocia (2)***			[28]
		*Halichondria panicea (2)*			[59,95]
		*Haliclona anonyma (1)*			[96]
		*Haliclona oculata (1)*			[97]
		*Haliclona tubifera (1)*			[98]
		***Hymeniacidon perlevis (5)***			[32,33,37]
		*Negombata magnifica (1)*			[99]
		*Pseudosuberites aff. Andrewsi (2)*			[100]
		***Tethya meloni (1)***			[36]
	HMA	*Agelas oroides (3)*	37.55 (20.15) [8.33–96.05]	72 (60.6) [11.41–253.79]	[63]
		*Geodia barretti (1)*			[66]
		***Geodia cydonium***	57.97 (5.41)	9.63 (1.68)	Present paper
Keratosa	LMA	*Dysidea avara (2)*	2925.9 (879.9) [2046–3805.8]	1.29 (0.62) [0.67–1.91]	[17,94]
	HMA	***Aplysilla rosea (1)***	133.83 (87.05) [2.38–537.25]	14.32 (2.07) [6.07–17.89]	[29]
		*Sarcotragus foetidus (3)*			[63]
		***Sarcotragus spinosulus (1)***			[35]
		*Spongia officinalis (1)*			[49]
Verongimorpha	HMA	*Chondrilla nucula (1)*	7.39 (1.82) [2.98–11.7]	11.36 (2.79) [2.99–14.15]	[65]
		*Chondrosia reniformis (3)*			[63]
		***Aplysina aerophoba***	75.25 (9.58)	5.44 (0.31)	Present paper

## 5. Conclusions

This work represents the first ex situ bioremediation trial with *A. aerophoba* and *G. cydonium* sponges against a multidrug-resistant *V. parahaemolythicus* strain. The opposing environmental conditions between the aquaculture system-related ambient origin and the starvation period could have caused oscula closure in some explants and highlights the importance of the environment of origin in ex situ experimentation. However, both species showed promising filtering and retention efficiencies, and it is likely that they are capable of digesting the potential pathogenic bacterium *V. parahaemolyticus*. The incubation period did not cause any visible stress or damage to the explants, suggesting no infectiveness of the antibiotic-resistant bacterium towards the sponge species. These resilience and bioremediation abilities, together with their proven rearing suitability in IMTA systems, have demonstrated their beneficial ecosystem role in a growing sector that seeks to reduce its impact on the environment. Finally, a particularly significant aspect of their bioremediation activity on bacteria lies in the sponges’ ability to help limit the spread of antimicrobial resistance in the case of multidrug-resistant strains, an emerging threat on a global scale.

While the current study provides insight into the filtration capacity of sponges and their potential role in bacterial bioremediation, further research is needed to assess their effectiveness in more complex environments, such as fish farm waters, where diverse microorganisms and organic matter coexist. Future experiments should also focus on quantifying the biomass of sponges required to maintain bacterial concentrations below harmful levels in mariculture systems. These studies would help validate the findings of this study and support the development of sponge-based bioremediation strategies in aquaculture.

## Data Availability

The data supporting the reported results of the study can be provided upon request by the first author.
